# Content development footprints for the establishment of a National Bioethics Committee: lessons from Nigeria

**DOI:** 10.1080/11287462.2021.1939548

**Published:** 2021-06-11

**Authors:** Chitu Womehoma Princewill, Ayodele Samuel Jegede, Adefolarin Malomo, Francis Chukwuemeka Ezeonu, Abdulwahab Ademola Lawal, Omokhoa Adeleye, Christie Oby Onyia

**Affiliations:** a National Biotechnology Development Agency, Umaru Musa Yar’Adua Expressway, Federal Capital, Abuja, Nigeria; b University of Ibadan, Oyo State, Nigeria; c Department of Neurological Surgery, University of Ibadan, Oyo State, Nigeria; d Department of Applied Biochemistry, Nnamdi Azikiwe University, Awka, Nigeria; e Civil Society for Ethics and Value Development Initiatives, Abuja, Nigeria; f University of Benin, Benin, Nigeria; g Godfery Okoye University, Enugu State, Nigeria

**Keywords:** Nigeria, Bioethics, National Bioethics Committee, National Bioethics Framework, National Bioethics Policy, UNESCO

## Abstract

Nigeria is experiencing, together with the rest of the world, consequences of relentlessly accelerating technological developments, in the contexts of relative lagging of developments in the Humanities, new discoveries in sciences and technological innovations, advances in medicine, changes in government policies and norms, rapid changes in the society, unhealthy practices in the area of food and agriculture, degradation of the environment as well as climate change. Furthermore, Nigeria as a Member State of UNESCO Bioethics is expected to have a National Bioethics Committee to enhance her participation in global concerns, as well as increase her opportunities to tap into global Bioethics resources. For this Committee to be established, the National Bioethics Framework and Policy Documents must be put in place. This paper discusses the rigorous process of developing the National Bioethics Framework and the National Bioethics Policy Documents as well as the need for a National Bioethics Committee in Nigeria.

## General introduction of bioethics and global perspective

The etymology and definition of “Bioethics” are well documented and discussed (Chadwick & Wilson, 2018; Faden & Kass, 1991; Sass, 2007). The same applies to the dynamics of its scope and emphases (De Vries et al., 2009; Have, 2007; Reich, 1994).

The two world wars, involving as they did, the apices of human advancement, were a worrisome jolt on the conscience of humanity, pointing to the residual and significant darkness in the content and methodology of human enlightenment (Neuberger, 2005; Shrestha, 2012; Shuster, 1997; Weindling et al., 2015). On the other hand, the courage of the moral pragmatic response to the way Politics and Science would be done point to the vastness of human possibilities. Both underscore the fact that ideologically, at various levels of human collectives, moral relativity may be upheld without bowing to ethical relativism by offering higher common or consensual lights of wisdom that would guide lower levels of conscience. In both Politics and Bioethics, Humanity continues its strides to improve but remains far from perfection. It is clear so far, that the intellectual temperament, background, training, and historical experience of individuals and groups vary and so do the challenges they face from their unique perspectives and their capacity to address same (Ahmad et al., 2015; Olweny, 1994).

In Bioethics, various guidelines were developed from earlier efforts on relevant Ethics (Shrestha, 2012; WMA, 2013). The rationally democratized responses to inadequate medical ethics contents and procedures, the institutionalized oversight of human research reflect the formalized lessons of history (Blackmer, 2010). The emergence of the Belmont Principles, the Development of Principalism by Beauchamp and Childress, the institutionalization, development and other impacts on Bioethics in the United States of America reflect the history of ideological Revolutions, Wars, Powerful Civil Rights advocacy movements, as well as the education and experiences of individuals and groups in the North-West of the World (Beecher, 2001; Brian & Cook-Deegan, 2017). The shades of these are also reflected as, for instance, the varying degrees of the rather masculine ethos of the American version as compared to those of continental Europe in say emphases on Individualism (Beecher, 2001; Brian & Cook-Deegan, 2017; Lenior, 1997).

In the global community, traditional institutions and professions have adopted but adapted the promises and powers of Bioethics (Capron, 2017; Elgharieb, 2015; Nairne, 1993; Nezhmetdinova, 2013). Since major challenges are more globalized than Capacity and Vulnerability, it is noteworthy that privileged bodies in the West and various World Organizations have encouraged and supported the development of Bioethics in the Developing World: Fogarty and UNESCO in Nigeria are examples.

Although the fine tuning of Bioethics continues globally, as illustrated by the greater attention to Climate and Environment, local and regional realities and orientations differ, and responses must reflect these (Faden & Kass, 1991; Gefenas & Lukaseviciene, 2017). Compounding post-modernism with the explosion of insight, technology and their various imbalances, Nigeria and similar countries have formidable potential challenges to healthy survival and flourishing. Although we lack the experience and social advantages of formal radical ideological revolutions and movements, which means that we cannot simply transplant Bioethics. We have a rich cultural heritage which with further cross-fertilization can stand us in good stead. Most nations consider it advantageous to have a pool of resources from which they can launch the addressing of emerging Bioethical challenges in their broadening tendencies, as, for instance, The Presidential Bioethics Commission of the United Sates (Brian & Cook-Deegan, 2017;De Vries et al., 2009; Capron, 2017). In responding to prompting, we consider it important to address challenges that have impaired our moral-response-capacity in the past.

### Need and relevance of a National Bioethics Committee

Politics, Economics, Natural Science and Technology have biological and psycho-social drives; the natural impropriety and imbalance that frequently attend their outcomes are not easy to foresee, determine, deter or remedy, even in the best of climes. Since the 1960s, countries have sought for ways to deal with the ethical issues emerging from new technologies arising from life sciences and medicine (Brian & Cook-Deegan, 2017; Faden & Kass, 1991). In 1966, in the USA, concerns were raised about research ethics on *in-vitro* fertilization, human cloning and brain science (Beecher, 2001; Callahan, 2012; Lock, 1990). Before then, there were series of unethical researches by the Nazis during the world war (Shrestha, 2012; Shuster, 1997; Weindling et al., 2015). The story of the Tuskegee syphilis study in the USA was another unique unethical research carried out by the United States Department of Public Health (Ogunbure, 2011). The bid to obviate unethical research gave rise to the international ethical codes and guidelines such as the Nuremberg Code, the Belmont Principle, the Helsinki Declaration, and Council for International Organization of Medical Sciences (CIOMS) etc. Over time, with rigorous processes and efforts, bioethics has become so central that Bioethics Committee has become an important organ of the United States Government and other countries (Elgharieb, 2015; Lock, 1990). Conventionally, most President of the United State of America has established a Presidential Advisory Committee on Bioethics (Capron, 2017).

In the late 1960s, Senator Walter Mondale's efforts in the Senate to have a Commission to look into ethical issues of new technologies met a brick wall (Brian & Cook-Deegan, 2017). It was argued that a bioethics committee would obstruct progress in science, including biological technologies and medicine, while others argued that such decisions should not just be technical but should also be subjected to public deliberations (Brian & Cook-Deegan, 2017). The debate continued until the Tuskegee Syphilis study was made open to the public. In 1974, President Gerald Ford signed the statute that established the National Commission for the Protection of Human Subjects of Biomedical and Behavioral Research (Brian & Cook-Deegan, 2017). Today, the National Commission is remembered for its unique work on research ethics. Senator Mondale was concerned that the biological technologies were racing ahead of the law and needed systematic and national exploration (Brian & Cook-Deegan, 2017). The national commission came up with a report which emphasized that “science was producing ideas and technologies that will not only change medicine, but ideas that will spill over into our culture”, (Brian & Cook-Deegan, 2017). From then on, National Bioethics committees began to spring up all over the globe (Gefenas & Lukaseviciene, 2017).

The United Nations Educational, Scientific, and Cultural Organization (UNESCO) is one international organ that has championed the development and establishment of countries’ National Bioethics Committees (Langlois, 2008: UNESCO, Establishing Bioethics Committee, 2005). UNESCO in 2005 adopted the Universal Declaration on Bioethics and Human Rights. The declaration states “that ethical issues raised by the rapid advances in science and their technological applications should be examined with due respect to the dignity of the human person and universal respect for and observance of human rights and fundamental freedom” (UNESCO, Establishing Bioethics Committee, 2005). The primary aim of this declaration is “to provide a universal framework of principles and procedures to guide member states in the formulation of the legislation, policies or other instruments in the field of bioethics". The declaration is binding on all UNESCO member states including Nigeria, which also formally assented to the declaration. In compliance with this, Member States are expected to have a National Bioethics Committee that is Independent, Multidisciplinary and Pluralistic at the national level (UNESCO, Establishing Bioethics Committee, 2005). For a National Bioethics Committee to be established in any Member State, in line with the 2005 Declaration on Bioethics and Human Rights, a National Bioethics Framework and a National Bioethics Policy Documents have to be developed in line with what is applicable to the Member State.

### Local perspectives and the importance of having a National Bioethics Committee in Nigeria

Life is full of risks and the urge to deal with these risks is a basic condition of our existence. Every day, new technologies and new products emerge all of which impinge on living pattern, human health and values. Every technological advancement come with its challenges and every scientific discovery or product has both good and bad aspects. Only man can determine the direction of scientific benefits, and to do this, advancement in every field of discipline need to be adequately assessed to ensure that they are safe, beneficent, non-maleficent, and justly applied, as well as environmentally and socially sustainable. In balancing scientific innovations, advances in medicine and societal concerns, a bioethics committee made up of experts in different fields is required.

A bioethics committee is a committee of individuals with critical frame of mind and a system of values that prepares them to lead in finding systematic ways to judge each new advancement, innovation and discovery as it evolves (UNESCO, Establishing Bioethics Committee, 2005). The committee provides a platform and bodies for ethical debates, analysis and policy development (UNESCO, Establishing Bioethics Committee, 2005). Bioethics helps to analyze problems in a sophisticated way that would make the work of policy makers and the society far more useful than it might otherwise be (UNESCO, Establishing Bioethics Committee, 2005).

The importance and urgency of the need for a National Bioethics Committee is not lost on developing nations, but they will usually need support in meeting such a need. Since 2009, Nigeria has convened series of National Bioethics Stakeholders Meetings, in a bid to develop a National Bioethics Framework and National Bioethics Policy Documents for the establishment of a multidisciplinary, independent, and pluralistic National Bioethics Committee (NABC) in Nigeria.

### NABC, NHREC, and other ethics committees in Nigeria

Health Research Ethics Committee has been in existence in Nigeria since the 1980s, but has been dormant with no activities or any mention of it during research (NCHRE, 2007). After the unethical clinical trial conducted by the pharmaceutical company Pfizer in 1996 in Kano, Nigeria, during an epidemic of meningococcal meningitis which left many children dead (Ezeome & Simon, 2008; Report of the investigation Committee, 2001). Nigeria saw a need to revisit the dormant Health Research Ethics Committee in the Federal Ministry of Health (Ezeome & Simon, 2008; Report of the investigation Committee, 2001). In 2007, the National Health Research Ethics Committee was established (NHREC). The NHREC in Nigeria became the apex body for health research ethics, and it is also responsible for, but not limited to ensuring adherence to guidelines that govern ethical research practices in Nigeria in order to ensure adherence to the protection of human research participants, as well as the use of animals in research (see the National Code of Health Research Ethics, 2007). It is also important to take notice of other various ethics committees in our research institutions and hospitals. These committees are trained, and supervised by the NHREC, and have specific mandates which are largely narrowed to research and medical ethics only. The proposed NABC is not intended to interfere with the mandate of such committees but will rather provide greater impetus to their mandates. The National Health Research Ethics Committee (NHREC) and other ethics committees in Nigeria do not attend to issues other than research and health ethics, and so this leaves a huge gap in dealing with ethical issues that may arise in other sectors of the country.

The National Bioethics Committee when established is expected to deal with a wide variety of challenges with a broad scope. An example is the proposed Infectious Disease Bill by the Nigerian National Assembly which is ladened with unethical proposals such as compulsory vaccination, forceful arrest of suspected carriers of an infectious disease, force acquisition of individual houses and lands if the government deems it fit for use without prior notice to the owners or occupants, and other open-ended propositions in sections of the document that completely defy the Declaration on Bioethics and Human Rights (The Guardian, 2020; Coves, 2020; see also the Nigerian Infectious Disease Act, 2020). If the National Bioethics Committee had been established, it would have prevented the presentation of the Infectious Disease Bill to the National Assembly, and this would have stopped the rancor and anger that rose within the citizens at the time. Since the committee is also expected to proffer advice to policy makers which will help to positively shape national policies, practices and foster debates around core ethical issues, the sponsors of the Bill would have first presented it to the National Bioethics Committee to look at and advice appropriately. The NABC when established will be guided by the National Bioethics Framework and Policy Documents as well as established international (and extant national) Codes, Declarations and Guidelines.

The NABC will be holistic and all-encompassing in its approach. It will advise the federal government and policy makers on all issues and aspects of our economy. Above all, it will act as an umbrella to all ethics committees in Nigeria where ethical issues affecting the country will be debated and solutions may be proffered. Although the NABC has the duty to also review research protocols, and ensure that human subjects are protected, the NHREC which is the apex body for such (see the National Code of Health Research Ethics, 2007) will solely be responsible for that. The NABC is important because of its holistic nature.

### Bioethical framework and policy

A critical evaluation of ethics usually concerns the action, the actor, the intention or the consequences. In considering how every citizen ought to embrace their duties, rights and obligations, we have found Immanuel Kant's duty ethics, known as the Kantian theory very helpful. This theory, which focuses on autonomy, stipulates that an actor should place a moral norm upon him or herself and obey it as an autonomous individual (Misselbrook, 2013). Citizens by this theory are expected to pay allegiance to the law of the country as enshrined in various extant regulatory documents and implied in the national anthem and pledge. Utilitarianism is of the view that an action is morally right if it results in pleasure, whereas it is wrong if it gives rise to pain (West, 2004). Achieving this depends on how individual actor or citizen exhibits the right virtue. It is expected that every citizen will base his or her action on the right virtues of selflessness, care, love and charity. So, virtue ethics suggests that citizens should take on morally good and responsible character as enshrined in the constitution and as ethically proper. These theories were important to our efforts. We have also adopted the principles of autonomy, maleficence, non-maleficence and justice according to Beauchamp and Childress (2009), in the contexts of the other theories mentioned above.

## Methodology

The drive for the establishment of a National Bioethics Committee in Nigeria began in 2009. In 2009, the United Nations Educational, Scientific, and Cultural Organization (UNESCO) on the invitation of Nigerian government through its “Assisting Bioethics Committee” (ABC) Project sponsored and organized the first National Bioethics Stakeholders Meeting in Abuja, Nigeria. This meeting brought together about 30 participants. Selections of participants’ were holistic to represent every sector of the economy, relevant institutions and organizations based on the UNESCO 2005 Declaration of Bioethics and Human Rights. The objective of the meeting was to provide the Nigerian government the information required towards the establishment of a national bioethics committee. At this maiden meeting, UNESCO explained to participants’ that their technical assistance would be provided to Nigeria after a Memorandum of Understanding is signed between the Nigerian National Commission for UNESCO and UNESCO. Also, at this meeting, the National Biotechnology Development Agency (NABDA) was nominated as the Focal Agency to drive this process because NABDA is a biotechnology agency and biotechnology raises major bioethical concerns.

In 2017, UNESCO again organized and sponsored the second National Bioethics Stakeholders Meeting in Akwanga, Nasarawa state, Nigeria. The aim of this second meeting was to ascertain the extent Nigeria had gone in establishing a National Bioethics Committee, prepare and review the draft of the memorandum of understanding to be signed with UNESCO. This second meeting was therefore a follow-up on the 2009 National Bioethics Stakeholders’ Meeting. It was agreed at this 2017 meeting that NABDA will be the secretariat for the proposed National Bioethics Committee with the Ministry of Science and Technology as the host ministry. At the end of the 2017 meeting, it was observed that the process of establishing a National Bioethics Committee in Nigeria was a tall dream because nothing new was done from 2009 to 2017 when the second meeting was held, and so, a road map was developed, and assignments were given to line ministries. In the roadmap, NABDA, as the Focal Agency for bioethics was mandated to produce the National Bioethics Framework and Policy documents needed for the establishment of a National Bioethics Committee. From the report of the 2017 meeting, it was obvious that a third National Bioethics Stakeholders meeting will be convened to draft the needed National Bioethics Documents.

In August 2018, the Federal Government, through the National Biotechnology Development Agency (NABDA) in collaboration with Nigerian National Commission for UNESCO (NATCOM-UNESCO) organized and sponsored the third National Bioethics Stakeholders Meeting in Abuja, Nigeria. The objective of the 2-day meeting that drew over 100 persons from different institutions, both government and private sector from all over Nigeria was to develop the first draft of the National Bioethics Framework and the National Bioethics Policy Documents. Building on cumulative deliberations from the previous meetings, and broad-based consultations, bearing in mind the current national realities including perceived challenges to ethical conducts in our society, as well as major factors of threats to life and national securities, social stability which directly and indirectly affect the understanding and implementation of Bioethical propositions, policies and their execution, the following priority areas were identified and agreed upon, for the development of the instruments: agriculture, defense & security, education, environment, health, and society. These areas demand special attention in the interest of learning, social dynamics, safety and protection of life and environment in the context of human dignity. This initial prioritization was done to enable focused deployment of interventions to important areas that can bring about early results in terms of heightened moral standards, attitudes and practices. The development will facilitate systematic programming towards ethical evolution by the relevant institutional arrangements in public and private sectors in a manner that can improve overall national ethos. Six Technical Working Groups (TWGs) covering the identified areas were set up at the August, 2018 meeting for the development of the respective sectoral frameworks and policies with particular attention to ethical principles and practices. Each technical working group had a chairperson and comprised of experts in the relevant fields. They were drawn from ministries, Agencies, Civil Society Organizations, Universities and Other Institutions. The meeting generated preliminary sketches and contents on the different areas for both the frameworks and policies. Specifically, each TWG defined their areas of focus and critically deliberated on them to generate ethical issues to be addressed.

Agricultural ethics working group identified land use and ownership, commodification of the common, farm animal welfare and handling, food safety, food security, impact of agriculture on the environment, consent, Labels and Consumer Choice, agribusiness as critical areas of national concern issues. Defense TWG discussed civil control of the military, professionalism versus careerism, individuality and ethical behavior, civilian–military relations, and monitoring and evaluation. In addition, cybercrime and security issues namely cyber bullying, phishing scam, identity theft scam, cyber stalking, and invasion of privacy were discussed. Educational Ethics TWG focused on cultural norms and practices, socialization, code of conduct, work ethics, and crime control. Environmental ethics TWG deliberated on six core topical issues namely solid waste management, desertification, climate change, biodiversity loss, erosion, soil pollution, air pollution, water pollution and human environment. Health TWG interrogated health system, traditional medicine, access to medicine, global health, organ transplantation, cloning, genetic medicine, biotechnology, biobanking, as well as neuroethics, public health and artificial intelligence. Societal ethics TWG addressed 11 broad thematic areas namely; leadership ethics, family ethics, work ethics, religious tolerance, communication ethics, sexual relationship, mode of dressing, gratification, political patronage, domestic violence, and national patriotism.

In October 2019, the six TWG chairpersons met in Abuja for 2 days to fine tune the bioethics working documents produced by the stakeholders in August 2019. Beyond the meeting, Technical Working Groups Chairpersons functioned individually and as a team to develop more robust and technical content with better differentiation for both documents in their respective areas. In the process, incremental drafts were drawn up. Following peer reviews and the technical support of senior staff of the National Biotechnology Development Agency (NABDA), a final draft was produced. By December 2019, the National Bioethics Framework and Policy Documents required for the establishment of a National Bioethics Committee were produced and submitted to the Federal Ministry of Science and Technology which is the host Ministry for presentation at the Federal Executive Council Meeting for the President's approval.

### Summary of the methodology

- Preliminary National Bioethics Stakeholders meeting was held in 2009 – organized by UNESCO to explain to Nigeria how to proceed on the establishment of a National Bioethics Committee.
- A follow-up meeting to the preliminary National Bioethics Stakeholders Meeting was held in 2017 – organized by UNESCO to check the progress after the preliminary meeting, and to start the process of developing the National Bioethics Documents.
- Nigeria organized the third National Bioethics Stakeholder's Meeting in 2019 – organized by the National Biotechnology Development Agency in collaboration with the Nigerian National Commission for UNESCO to develop the National Bioethics Framework and the National Bioethics Policy required for the establishment of a National Bioethics Committee:
  − Invited representatives from every sector of the country because bioethics cuts across every sector
  − Identified six key areas (Agriculture, Defense and Security, Education, Environment, Health, and Society) that needed national attention
  − Grouped the six key areas into Technical Working Groups (TWGs)
  − Selected Chairpersons for the TWGs using their educational qualifications and experiences
  − Collated ideas and contributions from all the six the Technical Working Groups
- Developed a raw skeletal National Bioethics Framework and Policy Documents
- Organized a meeting of the Technical Working Groups Chairpersons to fine tune the raw skeletal National Bioethics Framework and National Bioethics Policy Documents
- The First Draft of the National Bioethics Framework and National Bioethics Policy were finally developed.

## Discussion

As stated earlier, this paper is to discuss the rigorous process of developing the National Bioethics Framework and the National Bioethics Policy documents needed for the establishment of a National Bioethics Committee in Nigeria. The mandate of the proposed National Bioethics Committee will be to undertake all necessary procedures to generate appropriate decisions regarding cutting edge ethics challenges, as well as advise the Nigerian government, policy makers, and its relevant agencies on bioethical issues as they relate to policy guides on Agriculture, Defense and Security, Education, Environment, Health and Societal values and behavior. It is to actualize this that the National Bioethics Framework and National Bioethics Policy Documents were developed.

### Agriculture ethics

Agricultural ethics is an ethical practice that addresses ethical issues or questions arising from the interaction between production and distribution of food and fiber goods (Bert et al., 2016). Since farmers are the main producers of food for humanity, the well-being of citizens depend, to a large extent, on how we are able to eliminate hunger and malnutrition. This depends on how society is organized and is able to maintain social order and promote normative value in the agricultural sector. Generally, the ethical issues in agriculture globally, as well as in Nigeria are safety of food, treatment of animals, and use of chemicals; farm management, sustainability, trade agreement and sharing of information. While there are international guidelines for these issues, globally practical effort to translate most of the guidelines to framework and policies is still a challenge in many countries. For instance, many countries still lack national legislation, policies and guidelines for regulating the use of animals whether in research or business (Bert et al., 2016; Koyenikan, 2008; Nwagbo, 2000).

The thrust of the bioethical policy and framework is to ensure that the practice of agriculture and provision of food in Nigeria does not breach ethical principles of “do no harm” and benefit to ensure public good. In promoting the ethical agricultural practice there is the need to create a mechanism for balancing the interests of research and business and public good through proper engagement of food producers and consumers. Establishment of a National Bioethics Committee will fill in that gap.

### Defense and security sector ethics

Military ethics judges and justifies military actions from a moral point of view. It defines standards of good behavior for individual military personnel (as individuals and/or members of a group) and develops these standards. It has been argued that military ethics is a critical skill required by every service member which they should gain, develop, and enhance throughout their career. Ethics education provides people with the capacity to take informed decisions based on moral skills gained from ethics training (Emonet, 2018). According to Sgt. Maj. Florian Emonet, it provides individuals with the skill to engage with rhetorical questioning that help them make informed decisions about such things as use of weapon, for instance. The armed forces are organs of, and are accountable to the general civil society. The Nigerian nation places lethal weapons in the hands of individuals in the defense and security sector to hold in trust on behalf of the nation. To avoid misuse and fatalities, people entrusted with these weapons must be held on high moral and ethical values. The sanctity of life, dignity of the human being must be upheld at all times. Military operatives are oftentimes faced with threat to their own lives and must take split second decisions on survivability. Their mental state can determine the application of weapons inappropriately for self-interest. Thus the possibility of excessive use of force which can end up with civilian casualties may constitute blight in human civilization. If these dangers must be averted, ethics, values, professionalism and standard procedures of engagement in the defense and security sector is an imperative. The military must be held to strict code of conduct by the civil authority.

The new dimension of cyber security has implications on national defense and security. Regularly updated principled regulations must be instituted to control the activities of all actors who by virtue of widely versatile and available technology have become key players in the sector. Most worrisome is the phenomenon of Artificial Intelligence (AI), ability to create “thinking machines” raises a lot of ethical issues. The ethical questions relate both to ensuring that such machines do not harm humans and other morally relevant beings, and to the moral status of the machines themselves (Bostrom & Yudkowsky, 2014). Human dignity may be eroded through AI if individuals refuse to play their roles responsibly by performing their duties.

The emerging use of AI in the defense and security sector poses grave danger in terms of ethics and values. Removing decision-making from human being to machines creates a situation where human feelings, love and compassion is eliminated in matters of life and death. Human control must be kept in the loop of the decision system in order to retain compassion and accountability so central to the principles of ethics and values.

### Education ethics

The methodology and outcome of policy framework and policy content acknowledged the potential contributions of multiple stakeholders in the policy implementation process. The critical roles and responsibilities of all in this fundamental sector, especially of teachers in children's ethical formation were further emphasized.

This was corroborated by recent works of Iroegbu and Uyanga (2019) who empirically demonstrated the potentials of teachers’ professional ethics variables as predictors of the quality of educational output. Their use of Nigerian universities as study population is particularly instructive as the observed association appears even more likely at lower levels of education. In this regard, the growing recognition of the importance of ethics in the teaching profession from a more general perspective and the place of pupils and students as beneficiaries, by other authors like Gluchmanova (2015). These were reflected in Nigeria's educational ethics framework and policy. The potential of moral instructions in primary schools impacting discipline on the wider society, including families and work settings, is further discussed empirically by Ezema et al. (2017).

### Environmental ethics

Environmental ethics can be discussed with principles addressing specific environmental problems namely justice and sustainability; sufficiency and compassion; solidarity and participation. The use of environment is critical to the welfare and wellbeing of human beings. The determinant of environmental practice is the societal value. The value attached to the environment and the way people relate to it is determined by how it is perceived and understood (Hens & Susanne, 1998). Environmental value is driven by duty. Individual willingness to do the right thing is a motivation for an enhanced environment and the harmony man enjoys with the environment. The vulnerability of the environment is becoming more prominent in global consciousness, and in Nigeria. Rapid industrialization due to advances in science and technology has seriously and negatively impacted on the environment, not only of the industrialized but also the developing countries, since the world has become a global village (Barrow, 2018). This, therefore, emphasizes the importance of creating environmental ethics framework in Nigeria to enable the country join the league of Nations in the fight against “environmental crisis” caused by “spill-over” of industrialization.

### Health ethics

The Exercise in the Health areas revealed the availability of rich resources, high capabilities, various extant professional and research codes of Ethics and appropriate organs of implementations of oversight; with a history of growing complex challenges previously confronted. There are issues that are mainly (but not wholly) local, such as resource allocation at all levels of our health concerns and efforts in the nation. Principles that are ultimately at least supportive of Human Rights have been explored to proffer solution that involves openness, accountability, democratic approaches, among others. Traditional Medicine is another area with strong local flavors; the need for safe, efficient and respectful, just methods of appropriation and opportunity for further discoveries and development informed recommendations. The Health areas also had issues with advanced technology in which Nigerians are participating or in which we are becoming significant consumers such as Biotechnology, Nanotechnology, Artificial Intelligence, Neuro-ethics, and Genetic Medicine, in these, usual global discussions took cognizance of our peculiar sensitivities and vulnerabilities as a people in making recommendations. Issues of HIV, Organ- Transplantations, and Biobanking are not new but have newer challenges in the lights of internationalized efforts that require high level discusses, modulation, monitoring and interventions. Public Health Ethics has local coloration in the contexts of our culture and Human Rights and law enforcement records. Cloning is the one global concern that does not immediately appear problematic locally but is related to issues that do and unless we are proactive, avoidable challenges may occur. Our recommendations took cognizance of our Africanness and our spiritualties among others. Such are the strengths and weaknesses, opportunities, and threats that we tapped into in critically engaging these complex human, social and sometimes political and economically laden health area concerns.

### Societal ethics

Developing codes of ethical conduct where they do not exist and periodically reviewing ethical commitments to determine whether they are appropriate in the light of new knowledge and changes in circumstances are also part of the essential recommendations especially in this sector. We have noted how ideological and civil society revolutions and changes affect the definition, execution, monitoring and development of Bioethics in other climes. Such factors tend to create gaps in the successful implementation of Bioethics guidelines in developing societies and the addressing of these needs to be carried along in such places. This can be understood in the context of the social values. Societal value is a measure of individual behavior. According to De Villiers (1989), the basis of evaluation of human behavior is to be found in a system of values. Heyns opines that values are basic perceptions of the relative importance of our elements of existence. These perceptions always have to do with priorities, whereas norms are the functions which direct and evaluate human attitudes and actions. Norms of a society help greatly to sustain the values because norms reflect values and their enforcement.

Generally, societal ethics cannot be discussed in the absence of duty. This is because the content of duty is rooted in society being the basis of human existence. Therefore, duty, as action based phenomenon exists in the context of social web and forms the basis of culture and legal framework. Globally, the value a society upholds determines how the structure (education, health, politics, family, religion, etc.) will function as well as individual and collective behavior. This is dynamic across generations and sometimes raises concern among the elders (Akiwowo, 1983).

Social order is the hallmark of social organization yet no society has been able to achieve this in the history of mankind due to the dynamic nature of culture. Nigeria has evolved through series of life experiences, many of which had drastic effect on the orientation of her people and consequence for their wellbeing. Several sociological theories have been propounded to explain society but none is sufficient to do so. While these sociological theories (functionalist, action and conflict and their variants) have their origin in the western world, their adaptation compounds the situation in non-western culture (Agbaje & Adisa, 1988). Despite the existence of sociology as a discipline in Nigeria since 1960 the socio-political situation of the country has been begging for answer. Generally, the spade of socio-economic and political problems is aggravating while the quality of life of the people is reversing at high speed despite abundant resources in the country. Many past and present governments have put in place different intervention programmes such as “War Against Indiscipline”, Anti-Corruption plans, but none of these have been able to address the problem. This is because the programmes lack local content in terms of home grown bioethics policy and framework. To address the problem of a nation, it must be understood from the point of view of what the people value and the changing pattern of the value system (Osaat & Omordu, 2011). Although several scholars have worked on the changing value system in Nigeria, none of these works have attempted a holistic view of the problem (Agbaje & Adisa, 1988; Akiwowo, 1983; Opara, 2007).

## Conclusion

The background, challenges, and methods of achieving the production of the document with support, have been discussed. The implementation will likely be even more demanding. We shall need to remain committed, rigorous, cautiously creative, and critically open, in the next phases. It is hoped that all stakeholders will use the National Bioethics Framework and Policy Documents to drive debates and inform the infusion of more ethical approaches, contents and intentions in decision-making, further policy developments, legislations, reviews of regulatory instruments, implementations, judgment and behavior in their respective domains nationwide. Eventually, a culture of integrity, accountability and transparency should pervade our society and governance it in a manner that fosters public trust and global respect.

It is our view that considering the importance of the print, electronic and social media in shaping public opinions and morality, bioethicists should not only write books and publish scholarly articles, but should also take advantage of the print, electronic, and social media so as to be read and heard by a wider audience. This will also help to educate a lot of people on the importance of bioethics to the society and what it is all about. Having produced the National Bioethics Framework and Policy Documents, it is important for Nigeria to move towards establishing the National Bioethics Committee. A National Bioethics Committee cannot be ignored by any society that truly desires positive change.
